# First Breath Matters: Out-of-Hospital Mechanical Ventilation in Patients with Traumatic Brain Injury

**DOI:** 10.3390/jcm14238443

**Published:** 2025-11-28

**Authors:** Victoria Brinker, Aristomenis Exadaktylos, Wolf Hautz, Mairi Ziaka

**Affiliations:** 1 Department of Emergency Medicine, Inselspital, University Hospital, University of Bern, 3010 Bern, Switzerland; 2 Department of Internal Emergency Medicine, Thusis Hospital, 7430 Thusis, Switzerland; 3 Academic Department of Emergency Medicine, School of Medicine, University of Cyprus, 1678 Nicosia, Cyprus

**Keywords:** cerebral autoregulation, hypercapnia, hyperoxemia, hypocapnia, hypoxemia, mechanical ventilation, preclinical setting, traumatic brain injury

## Abstract

Invasive mechanical ventilation (MV) is often a lifesaving intervention in patients with traumatic brain injury (TBI) to optimize gas exchange and prevent secondary brain injury, thereby avoiding the deleterious effects of both hypoxia and hyperoxia, as well as hypocapnia and hypercapnia. However, MV in these patients represents a unique clinical challenge, as it must take into account multiple parameters, including cerebral autoregulation and autoregulatory reserves, brain compliance, cerebral dynamics such as intracranial pressure (ICP), cerebral perfusion pressure (CPP), and cerebral blood flow (CBF), as well as systemic hemodynamics and respiratory system mechanics. Moreover, the detrimental effects of MV on extracranial organs and systems are well established, with the lungs being the most vulnerable, particularly when non-protective ventilation strategies involving high tidal volumes (TV) and inspiratory pressures are applied. Currently, the optimal ventilation approach in patients with TBI, with or without LI, remains incompletely defined. While protective ventilation practices are recommended for a large number of critically ill patients, their application in individuals with acute brain injury (ABI) may adversely affect cerebral and systemic hemodynamics, as well as brain physiology, potentially leading to secondary damage and poor clinical outcomes. Because the consequences of TBI, such as secondary brain damage and lung complications, begin shortly after the primary event, the role of prehospital MV in these patients is crucial. However, existing data from the out-of-hospital setting are scarce. Thus, in the present review, we aim to summarize the available evidence on MV in patients with TBI, with an emphasis on the prehospital setting.

## 1. Introduction

With an annual global incidence of 50 million cases, TBI, defined as a nondegenerative, noncongenital injury to the brain occurring via an external physical force, is the leading cause of death and disability among individuals under 40 years old [1,2,3,4]. Cerebral hypoxia, which acts as a secondary insult that worsens the primary brain injury, is a significant risk for patients with TBI, stemming from a combination of factors including vascular damage that reduces cerebral blood flow (CBF), cerebral edema leading to intracranial hypertension which compresses blood vessels and further limits oxygen delivery, and systemic conditions such as hypotension, anemia, and hypoxemia [5,6]. Indeed, adequate CBF and sufficient arterial oxygen content (CaO_2_) are two essential conditions for ensuring the availability of oxygen to the brain (DO2) [5,7]. Indeed, in neurocritical care, maintaining normal partial pressures of arterial oxygen (PaO_2_) and preventing hypoxia are crucial. The IMPACT study database, which includes over 9000 TBI patients enrolled in randomized controlled trials and series since the 1980s, has confirmed the significant impact of hypoxemia on mortality and poor outcomes [8]. Regarding this issue, it could be hypothesized that increasing the fraction of inspired oxygen (FiO_2_) could be a practical approach to prevent hypoxemia and cerebral tissue hypoxia. Nevertheless, even with supranormal levels of PaO_2_ (normobaric hyperoxia), occult cerebral hypoxia can still persist [9]. On the other hand, oxygen concentrations reaching nonphysiological hyperoxemic ranges may be associated with harmful effects. A retrospective study comparing mortality rates and PaO_2_ levels in mechanically ventilated intensive care unit (ICU) patients reported a U-shaped relationship, with increased mortality rates noted at low and high PaO_2_ levels [10]. Both clinical and experimental studies have demonstrated that exposure to hyperoxia is linked to lung injury (LI), inflammation, impaired hemodynamics, reduced oxygen consumption, and increased mortality in a dose-dependent manner [11]. Thus, for patients with brain injury, it is recommended to maintain PaO_2_ levels between 80 and 120 mm Hg according to the European Society of Intensive Care Medicine (ESICM) [12]. While clinicians generally acknowledge the claim to target normoxia in trauma patients [13], hyperoxemia is common in trauma and associated with worse outcomes [14].

Although MV is a lifesaving tool frequently used in critically ill patients, its application in the general ICU population, particularly with the use of high TV and inspiratory pressures, has been shown to overstretch the alveoli, leading to ventilator-induced lung injury (VILI) [15,16,17]. While protective MV with low TV and positive end-expiratory pressure (PEEP) is well-documented to reduce mortality in acute respiratory distress syndrome (ARDS) patients, its application to all critically ill patients remains uncertain [18]. Furthermore, it is crucial to consider that this protective ventilation strategy can lead to self-inflicted lung injury [18], hypercapnia [19], and hypoxemia [20] with significant consequences, particularly in patients vulnerable to hypercapnia and hypoxemia, such as neurocritically ill patients and those with cardiac arrest [21,22]. Indeed, MV in ABI patients presents several unique challenges. Ventilator settings must be tailored to mitigate potential adverse cerebrovascular effects, consider the impact on intracranial circulation, account for cerebral autoregulatory reserve, and maintain brain compliance in order to avoid intracranial hypertension and reduced CBF [23]. Even minor increases in arterial partial pressure of carbon dioxide (PaCO_2_) in ABI patients are associated with cerebral vasodilation, leading to intracranial hypertension and increased cerebral blood volume (CBV) [19,23,24]. Traditionally, ABI patients were ventilated with high TV because hypocapnia has been observed to reduce ICP and maintain normal ICP [23]. However, hyperventilation and resulting hypocapnia can be detrimental for ABI patients, especially within the first 24 h after the injury, when cerebral homeostasis is severely compromised [24,25,26]. Moreover, given that PEEP can elevate intra-thoracic pressure and potentially impair central venous return, leading to increased intracranial pressure, the appropriate level of PEEP in ventilatory settings is a concern that has not been well-documented [27], particularly in the prehospital setting.

Lung complications such as pneumonia, LI/ARDS, and pulmonary mechanics deterioration typically occur within 2 days after TBI [15,28,29], emphasizing the critical importance of effective MV management immediately after intubation. This highlights the essential role of appropriate ventilator management in prehospital and emergency department treatment. However, unlike ICU or intraoperative settings, MV in these areas has historically received minimal research, trainee education, and clinical emphasis [30]. Therefore, the present work aims to explore prehospital MV settings and the impact of oxygenation on outcomes for patients with TBI. It will examine parameters such as TV, PEEP, FiO_2_, and the levels of PaO_2_ and PaCO_2_, evaluating their influence on survival rates, neurological recovery, and complications, with the goal of improving medical care in TBI patients.

## 2. Methods

A comprehensive, non-systematic literature search was conducted using PubMed to identify relevant studies on mechanical ventilation in patients with TBI, with an emphasis on the prehospital setting. The search terms included “traumatic brain injury”, “acute brain injury”, “mechanical ventilation”, “cerebral perfusion pressure”, “intracranial pressure”, “prehospital”, “out-of-hospital”, “positive end expiratory pressure”, “tidal volume”, “lung protective ventilation”, “multimodal neuromonitoring”, “cerebral autoregulation”, “hypoxia”, “hypoxemia”, “hyperoxemia”, “hypocapnia”, “hypercapnia”, and “critical care”. Boolean operators (AND, OR) and truncations were used to refine and optimize search results. The search focused on articles published in English over the past 20 years (2005–2025) to extract contemporary evidence, as understanding of cerebral autoregulation, MV, and overall neurocritical care management has advanced significantly during this period. Classical articles published before 2005 were also included to provide historical context and a comprehensive understanding. Additionally, manual screening of references from selected studies was conducted to identify further relevant articles and grey literature.

## 3. Cerebral Autoregulation in TBI

The term cerebral autoregulation denotes the complex homeostatic processes that regulate both local and global blood flow across a variety of conditions and changes in cerebral perfusion pressure (CPP), thereby ensuring the brain’s metabolic demands are met [31,32,33]. Unlike other organs, the brain is enclosed within an unyielding bony cavity with a fixed intracranial volume, occupied by brain tissue, blood, and cerebrospinal fluid (CSF), where changes in the volume of one component directly affect ICP, such that an increase in one element (brain, CSF, arterial or venous blood) leads to a compensatory decrease in another to maintain stable ICP (Monro-Kellie Doctrine) [32,34,35]. Since CPP is determined by the gradient between mean arterial blood pressure (MAP) and ICP, the maintenance of stable CBF across a range of MAP is mediated by reductions in cerebrovascular resistance and adjustments in the diameter of cerebral arteries through myogenic, neural, endothelial, and metabolic mechanisms [36,37,38]. Indeed, it has been convincingly demonstrated in recent years that smooth muscle cells of the cerebral vasculature can constrict or dilate in response to changes in vascular wall tension through calcium-dependent processes, which immediately result in changes in vessel diameter [39,40]. Moreover, in response to alterations in CBF, transmural pressure, and shear stress, endothelial mechanoreceptors stimulate the release of vasoactive agents, including nitric oxide (NO), endothelium-derived hyperpolarizing factor (EDHF), eicosanoids, prostacyclin, and endothelin-1. These factors regulate vascular tone, with NO and prostacyclin promoting vasodilation and increasing CBV, while endothelin-1 and 20-hydroxyeicosatetraenoic acid (20-HETE) induce vasoconstriction [38,40,41,42,43]. Experimental studies in models of TBI indicate that these protective mechanisms are impaired in individuals with TBI, leading to insufficient constriction in response to intraluminal pressure elevations and, consequently, high intraluminal pressures and brain edema [44]. Moreover, through both afferent and efferent nerve endings, including α-adrenergic nerves, cerebrovascular vessels are able to modify cerebrovascular tone, vessel diameter, and CBF [45,46,47].

Traditionally, cerebral autoregulation is described as a sigmoid, triphasic curve, with a lower limit of steady CBF, an upper limit, and a plateau between these limits, representing static cerebral autoregulation. Indeed, in a classical study, Lassen (1959) analyzed 7 studies and 11 distinct patient populations and found that CBF remained stable despite fluctuations in arterial blood pressure between 60 and 150 mm Hg [32]. Certainly, while CBF remains relatively stable at the plateau level, beyond these thresholds, the pressure–flow relationship shifts to a more passive state, leading to alterations in CBF—decreasing below the lower limit of autoregulation (LLA) and increasing above the upper limit of autoregulation (ULA)—which can potentially cause secondary brain injury, including hypoperfusion and associated hypoxic damage or hyperemia resulting in cerebral edema and BBB disruption, respectively [31,48]. However, Klein and co-authors (2021) recently presented a quadriphasic curve in an experimental model of cerebral autoregulation, including two different ULA. In this model, cerebral autoregulation progressively fails, starting at the smallest arterioles and extending to larger ones, until all arterioles lose their ability to resist rising CPP, at which point blood flow becomes entirely pressure passive [49]. Cerebral autoregulation is impaired in patients with different severities of TBI and is strongly associated with unfavorable long-term outcomes due, among other factors, to elevation of ICP, ischemia, and hyperemia [50,51]. Importantly, disrupted cerebral autoregulation in TBI can occur even at normal ICP and MAP values [52].

Dynamic cerebral autoregulation refers to the response of the cerebral vasculature to alterations in arterial blood pressure, occurring within seconds to minutes, for example, during changes in body position or physical activity. Nevertheless, CBF varies significantly with the degree of fluctuation, depending on the oscillation frequency. This cerebrovascular response to changes in arterial blood pressure is further influenced by PaCO_2_ levels, with response times ranging from ~4 s in hypocapnia to ~7 s in hypercapnia [53]. Moreover, in the course of time, advances in understanding cerebral physiology have highlighted the dynamic rather than static interactions between intracranial compartments, emphasizing venous circulation—particularly outflow—as a cornerstone of ICP regulation. Extracranial factors, including alterations in intrathoracic and intra-abdominal pressures or impaired venous drainage due to jugular vein compression, can lead to intracranial hypertension even in the absence of a brain lesion causing a mass effect [54].

## 4. Gas Exchange Alterations and Cerebral Homeostasis in Traumatic Brain Injury

**Figure 1:**
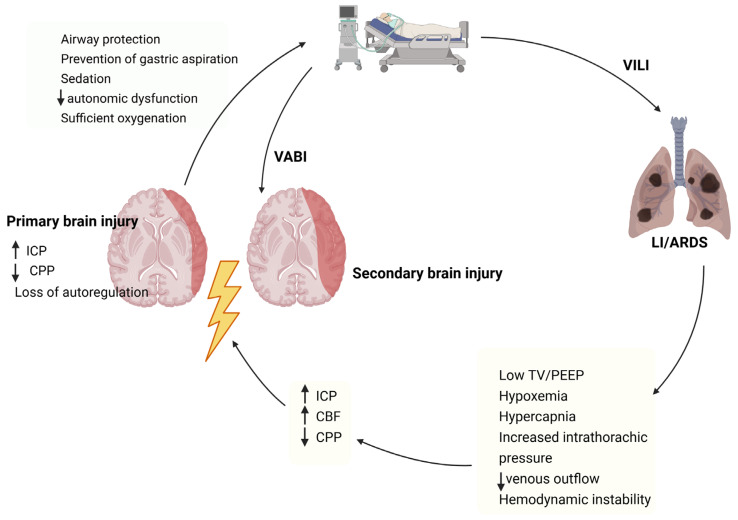
Simplified schematic representation of the pathophysiology of secondary brain injury in patients with TBI involving mechanical ventilation and the development of LI/ARDS. ARDS: acute respiratory distress syndrome; CBF: cerebral blood flow; CPP: cerebral perfusion pressure; ICP: intracranial pressure; LI: lung injury; PEEP: positive end expiratory pressure; TBI: traumatic brain injury; TV: tidal volume; VABI: ventilator associated brain injury; VILI: ventilator induced lung injury complications—such as neurogenic pulmonary edema, ventilator-associated pneumonia (VAP), and ARDS—as well as prolonged cognitive dysfunction and increased mortality compared with critically ill patients without brain injury [15,66].

Cerebral blood vessels smaller than 50 μm exhibit “CO_2_ reactivity,” altering their diameter in response to changes in PaCO_2_ levels; they dilate with hypercapnia (PaCO_2_ > 44 mm Hg) and constrict with hypocapnia (PaCO_2_ < 35 mm Hg), with this vascular activity occurring between PaCO_2_ levels of 20 and 60 mm Hg, though the upper limit remains poorly defined, and the CBF response to PaCO_2_ changes follows a sigmoid curve [55,56]. Exceeding 80 mm Hg in PaCO_2_ leads to vasodilation, enhancing CBF by approximately 100–200% and stimulating catecholamine release and metabolic activity (Figure 1), whereas during hypocapnia, each 1 mm Hg reduction in PaCO_2_ results in a 3% decrease in CBF, with PaCO_2_ levels between 20 and 25 mm Hg correlating with a 40–50% reduction in CBF elevating the risk of cerebral ischemia and hypoxia, especially in the acute phase of TBI [56,57,58,59]. Adding to the complexity, the interactions between hypocapnia and hemodynamics become more intricate in patients undergoing positive pressure ventilation, sedation, and experiencing hypovolemia [60]. However, the data regarding the relationship between PaCO_2_ and cerebral oxygenation/metabolism or clinical outcomes are scarce and conflicting, and mainly originate from research in the ICU. An older study highlights that following hyperventilation, there was a significant reduction in cerebral blood flow observed using Positron emission tomography (PET) scans, accompanied by an increase in the volume of severely hypoperfused tissue, corresponding to a decrease in PaCO_2_ from 4.8 to 3.9 kPa [61]. However, a study using a similar PET technique did not find any alterations in oxygen metabolism among two groups of TBI patients who received varying levels of hyperventilation [62]. In terms of clinical outcomes, Citerio et al. (2021) found that PaCO_2_ is lower in patients with ICP monitoring, especially if ICP is increased, but they did not find evidence of worse outcomes in patients treated in centers that more frequently utilize profound hyperventilation [63]. Similarly, a prospective study from Switzerland examining patients with nonpenetrating TBI did not observe detrimental effects of hypocapnia on cerebral metabolism [64]. However, a recent study demonstrates a U-shaped association between the first 24-h PaCO_2_ levels and in-hospital mortality in patients with ABI [65].

Although the recent recommendations of the ESICM suggest a consensus that the optimal range of PaCO_2_ in neurocritically ill patients is between 35–45 mm Hg [12], evidence in patients with cerebral injury secondary to cardiac arrest indicates that mild hypercapnia may be beneficial. This is due to its ability to increase CBF [67,68,69] and its anticonvulsive [70] and anti-inflammatory properties [71]. Indeed, cerebral vasodilation caused by hypercapnia can enhance CBF and improve brain oxygenation, potentially offering benefits. A recent secondary analysis of the ENIO study (Extubation Strategies in Neuro-Intensive Care Unit Patients and Associations with Outcomes; registration number NCT03400904) reveals a U-shaped relationship between PaCO_2_ and in-hospital mortality, with only severe hypocapnia and hypercapnia linked to a higher likelihood of in-hospital mortality and important variations among different subgroups of ABI patients [72,73]. Nevertheless, in extreme cases, hypercapnia may disrupt CBF regulation, which could worsen brain edema and lead to secondary brain injury and poor prognosis [74,75].

## 5. Effects of Mechanical Ventilation on Cerebral Autoregulation

Patients with TBI very frequently require endotracheal intubation and MV for airway protection, prevention of gastric aspiration, adequate sedation to manage anxiety, restlessness, pain, and behavioral dysregulation, to improve cerebral metabolism, to limit autonomic dysfunction [15,66,76], and to ensure sufficient oxygenation to prevent secondary brain injury (Figure 1) [33,77]. However, given the complex interactions among the brain, respiratory, and circulatory systems, MV in patients with ABI is particularly challenging, as it can affect key cerebral parameters—including ICP, CPP, and CBV—potentially impairing clinical outcomes [53]. Additionally, it is well established that MV can cause LI through overstretching, repeated alveolar collapse and re-expansion with each breath, and mechanotransduction—the conversion of mechanical forces into biological signals—triggering local and systemic inflammatory cascades (Figure 1) [21,78].

Protective MV with low tidal volume TV and PEEP reduces mortality in ARDS patients, but its applicability to all critically ill patients remains uncertain by potentially leading to self-inflicted lung injury [18], increased intrathoracic pressure, reduced venous return [79], hypercapnia [19], intracranial hypertension, and detrimental alterations in cerebral hemodynamics [15], potentially leading to secondary brain injury [80]. These brain–lung interactions in mechanically ventilated patients with TBI are of major importance, as neurocritically ill patients with brain injury generally require prolonged MV due to higher rates of respiratory.

Nevertheless, despite the lack of strong evidence, a lung-protective ventilation strategy has been suggested for all patients with ABI and normal ICP [12], with cautious titration of TV, PEEP, driving pressure, and inspired FiO_2_ [81].

PEEP can influence cerebral autoregulation and the associated intracranial dynamics, including ICP and CPP, in patients with ABI, with its effects varying across individuals. Patients with intact cerebral autoregulation generally demonstrate stable responses to moderate PEEP, in contrast to those with impaired autoregulation [82]. Recently, a prospective crossover study of 30 mechanically ventilated patients with ABI but without lung injury, randomized to receive either low (5 cm H_2_O) or high (12 cm H_2_O) levels of PEEP, found no differences in cerebral autoregulation capacity or brain compensatory reserve. However, intracranial hypertension was observed in nearly 25% of participants, leading to interruption of the intervention for safety reasons [83]. These findings are in line with those of Giardina et al. (2023) in patients with ABI receiving MV, highlighting that slow and gradual elevations in applied PEEP did not influence cerebral autoregulation, cerebral oxygenation, ICP, or CPP to levels requiring therapeutic intervention [84]. The aforementioned findings are further supported by experimental models investigating the effects of PEEP on ICP and cerebral autoregulation in individuals with normal ICP and without LI, in both prone and supine positions, using the pressure reactivity index (PRx). These studies found that increases in PEEP did not affect cerebral autoregulation but led to elevations in ICP [85].

Finally, the impact of TV on cerebral autoregulation in brain-injured patients is primarily mediated by alterations in PaCO_2_ levels. Low TV is associated with hypercapnia, leading to vasodilation and increased intracerebral blood volume, whereas higher tidal volumes are associated with hypocapnia and cerebral vasoconstriction, resulting in reduced CBF, decreased ICP, and transient improvements in cerebral autoregulation. However, excessive and prolonged reductions in PaCO_2_ may increase the risk of secondary ischemic injury [86,87].

## 6. Ventilatory Strategies in TBI

Despite being a cornerstone of neuroprotective management in patients with TBI, MV primarily serves to maintain adequate oxygen delivery and regulate cerebral hemodynamics by controlling PaCO_2_ levels, thereby preventing secondary brain injury [12,88]. However, its application may exert detrimental effects due to multifactorial interactions among the intrathoracic, intracranial, and cardiovascular systems [12,89]. In particular, elevations in intrathoracic pressure—such as those associated with the application of PEEP—may reduce cerebral venous outflow, leading to increased ICP and hemodynamic instability that impairs CPP. Conversely, hypercapnia secondary to alveolar overdistension may result in cerebral vasodilation [84]. Consequently, the optimal ventilation strategy for patients with TBI remains largely unclear, as these patients are commonly excluded from studies evaluating lung-protective ventilation settings, and high-quality data regarding the ventilatory management of moderate to severe TBI are lacking [12,53]. Given the importance of preventing secondary insults and the well-established evidence that hypoxic and hypotensive events occur shortly after TBI in the prehospital setting and are independently associated with prognosis, the need to initiate neuroprotective strategies in the early phase of TBI is undisputed; however, the existing data remain scarce [90,91,92]. On the other hand, lung-protective ventilation is of particular importance in this patient population, given the high incidence of ARDS—occurring in up to 20% of cases—and the systemic inflammatory cascades, neuroinflammation, and catecholamine surge that occur shortly after TBI, making the lungs especially vulnerable to secondary damage and VILI [15,93].

## 7. Goals of PaO_2_ and PaCO_2_

The detrimental effects of hypocapnia on outcomes in TBI patients were recognized over three decades ago, when Muizelaar et al. (1991) reported significantly worse neurological outcomes at 3 and 6 months in patients with severe and sustained hypocapnia (PaCO_2_ 25 ± 2 mm Hg) compared with those with nearly normal PaCO_2_ levels (35 ± 2 mm Hg) [94]. Hypocapnia causes constriction of the cerebral arterioles, leading to reductions in CBF and CBV, altering brain metabolism, increasing neuronal excitability, and promoting neurotoxicity [56,58,59,95,96]. Alterations in brain metabolism occur early after the onset of hypocapnia (at approximately PaCO_2_ levels of 25 mm Hg), typically within the first 24–36 h, as evidenced by increased extracellular concentrations of glutamate and lactate in the brain, as measured by microdialysis [97]. Moreover, the systemic effects of hypocapnia on extracerebral organs are well established. In addition to the well-documented risk of LI, hypocapnia and hyperventilation can induce right ventricular dysfunction due to elevations in intrathoracic pressure—an effect particularly relevant in hypovolemic states, where venous return and cardiac output are reduced. Furthermore, hypocapnia has been associated with myocardial ischemia, pulmonary vasodilation and bronchoconstriction, and impaired renal arterial blood flow [58]. Moreover, a retrospective study including 9660 patients with ABI, including those with TBI, demonstrated a U-shaped association between arterial PaCO_2_ levels during the first 24 h after injury and mortality, with normocapnia (35–45 mm Hg) being associated with better clinical outcomes [65]. These findings are consistent with those of Robba et al. (2024) [72], a secondary analysis of the ENIO trial, a multicenter prospective observational study including 1476 patients with ABI, including those with TBI. This analysis also demonstrated a U-shaped association between PaCO_2_ levels and in-hospital mortality, suggesting that mortality increases with the severity of both hypercapnia and hypocapnia [72]. Based on existing evidence, a consensus panel of international experts strongly recommends maintaining PaCO_2_ levels between 35 and 45 mm Hg in ABI patients without intracranial hypertension. In addition, they weakly recommend short-term hyperventilation in cases of brain herniation, while no recommendation could be made regarding the use of short-term hyperventilation for the management of intracranial hypertension [12].

**Figure 2:**
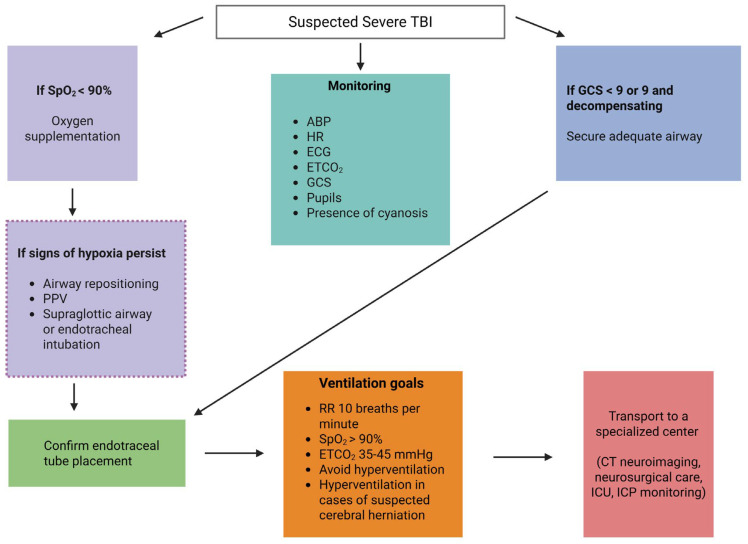
Prehospital oxygenation management in patients with suspected severe TBI [103,104]. ABP: arterial blood pressure; CT: computed tomography; ECG: electrocardiogram; ETCO_2_: end-tidal carbon dioxide; GCS: Glasgow coma scale; ICP: intracranial pressure; ICU: intensive care unit; PPV: positive pressure ventilation; SpO_2_: oxygen saturation.

The available evidence regarding prehospital PaCO_2_ targets is scarce. Nevertheless, given that the metabolic consequences of hypocapnia occur immediately after the traumatic event and that prehospital hypocapnia is associated with worse clinical outcomes and increased mortality [97,98,99], careful attention should be given to prehospital PaCO_2_ management. The Excellence in Prehospital Injury Care (EPIC) Study, a large study including 21,852 patients with TBI—of whom 4014 required intubation—defined a PaCO_2_ target of 40 mm Hg (range 35–45 mm Hg) under continuous end-tidal carbon dioxide (ETCO_2_) monitoring to avoid hyperventilation and reduce the incidence of hypocapnia [100]. Based in part on the findings of the EPIC study, recent guidelines on the prehospital management of TBI strongly recommend maintaining normal PaCO_2_ levels, i.e., ETCO_2_ of 35–40 mm Hg [101]. Nonetheless, in the presence of clinical signs of brain herniation—such as Cushing’s triad, progressive decline in level of consciousness, extensor or flexor posturing, or pupillary abnormalities including mydriasis, loss of light reactivity, and anisocoria—the expert panel strongly recommends therapeutic hyperventilation with a target ETCO_2_ of 30–35 mm Hg, monitored using capnography (Figure 2) [101,102].

It is now widely accepted that both hypoxemia (PaO_2_ < 60 mm Hg) and hyperoxemia—although variably defined across studies—are associated with poorer neurological outcomes and increased mortality [105,106,107]. A systematic review and meta-analysis by Hirunpattarasilp et al. (2022) [107], including 11,757 patients with ABI (2307 with TBI), found that hyperoxemia was associated with worse neurological outcomes, with a trend toward poorer outcomes at higher PaO_2_ levels. However, in the TBI subgroup, this association did not reach statistical significance [107]. Furthermore, a secondary analysis of two multicenter, prospective, observational cohort studies (CENTER-TBI and OzENTER-TBI) reported that exposure to high arterial oxygen levels or excessive supplemental oxygen was independently associated with increased 6-month mortality. The authors, however, noted that this association might partly reflect the use of higher FiO_2_ in patients with greater injury severity and physiological decline [106,107]. Moreover, it is well established that oxygenation with high oxygen concentrations may lead to cerebral and coronary vasoconstriction, direct lung damage, and atelectasis [108,109]. These findings are further supported by experimental research highlighting that hyperoxia may cause lipid peroxidation and neutrophilic activation in brain regions such as the hippocampus and cerebellum, increase oxidative stress and is associated with mitochondrial dysfunction in various brain structures, cause neuronal damage, induce the production of reactive oxygen species and the generation of inflammatory reactions, resulting in structural and functional alterations of the brain—effects partly dependent on the concentration of supplemental oxygen and the duration of exposure [110,111,112,113]. Finally, it is important to emphasize that even in cases of normobaric hyperoxia, hidden cerebral hypoxia may be present [5]. A recent randomized trial of severely injured trauma patients compared a liberal versus a restrictive oxygenation strategy during the first eight hours after major trauma starting in the prehospital phase in 1508 patients and found both strategies to be safe, while less atelectasis occurred in the restrictive group [114]. A subgroup analysis of TBI patients from this trial is underway.

Preventing hypoxia is one of the cornerstones in the management of severe TBI, as more than 90% of patients with fatal TBI have shown posttraumatic ischemic findings at autopsy [115]. The supply and delivery of oxygen to the brain depend on numerous physiological factors, including insufficient oxygen provision and impaired oxygen metabolism at the cellular level [12,116]. In their classical meta-analysis of seven Phase III randomized control trials (RCTs), McHugh and co-authors (2007) highlighted that hypoxemic patients with moderate to severe TBI had significantly worse 6-month neurological outcomes [8]. The prevention of hypoxia, defined as PaO_2_ ≤ 60 mm Hg, is vital immediately after severe TBI, as pre-hospital hypoxemia has been shown to be independently associated with increased mortality and worse clinical outcomes [117]. Unfortunately, studies in the prehospital setting have demonstrated that hypoxia is common in patients with TBI and is associated with a threefold increase in the likelihood of death, with an elevated risk observed even after a single episode of low oxygen saturation [118,119]. Indeed, a recent study including 1210 patients with TBI found that more than one-quarter had moderate prehospital hypoxia (oxygen saturation, SpO_2_ 80–93%), while nearly 10% had severe hypoxia (SpO_2_ < 80%), with worse neurological outcomes increasing significantly with the degree of hypoxia [120]. A retrospective study including 3420 patients with moderate to severe TBI demonstrated that PaO_2_ levels < 110 mm Hg upon arrival at the emergency department were associated with increased mortality and worse clinical outcomes [121]. Given the detrimental effects and poor prognoses associated with both hypoxemia (commonly defined as PaO_2_ < 60 mm Hg) and hyperoxemia (with its definition still debated, typically PaO_2_ > 150 or 300 mm Hg), a target SpO_2_ of 94–96% and a PaO_2_ level of 80–120 mm Hg are recommended [53,77]. In prehospital care, it is strongly recommended that patients with TBI be continuously monitored using pulse oximetry to maintain SpO_2_ levels > 90% through continuous supplemental oxygen administration and optimal airway positioning [101]. However, it should be noted that procedures and ventilation targets vary worldwide. In the United States, as previously mentioned, an SpO_2_ target above 90% and an ETCO_2_ of 35–45 mm Hg is recommended, whereas internationally, the World Society of Emergency Surgery recommends maintaining SpO_2_ > 94% and PaCO_2_ between 35 and 38 mm Hg (Figure 2) [101,103].

## 8. Tidal Volume

In recent years, it has become increasingly evident that lung-protective MV—characterized by low TV and the application of moderate PEEP—in critically ill patients without ABI is associated with improved prognosis compared with ventilation strategies using higher TV and lower PEEP levels [122,123,124,125], particularly due to reductions in cyclic recruitment–derecruitment and TV-related hyperinflation [126]. The beneficial effects of protective MV with low TV have also been proposed for patients with ABI [127]. However, the use of lower TV is associated with moderate hypercapnia, as suggested by research comparing lower versus higher VT in critically ill patients without ARDS [124]. Nonetheless, hypercapnia represents an important issue in patients with acute intracranial pathologies, including TBI, as it causes vasodilation of the cerebral vasculature, decreases vascular resistance, and increases CBF, thereby leading to ICP elevation [128,129]. Besides the effects of PaCO_2_ on the cerebrovascular endothelium, the carbonic anhydrase pathway is a key physiological contributor to hypercapnia-associated cerebral vasodilation by decreasing pH in the CSF, resulting in reduced tone of vascular smooth muscle cells [130]. Recently, a secondary analysis of the ENIO study [73,131], conducted across 18 countries and including 1510 patients with ABI (the majority being TBI patients), compared MV with TV ≤ 8 mL/kg predicted body weight (PBW) and >8 mL/kg PBW over the first 7 days of MV, reporting mortalities of 40.2% and 59.7%, respectively. In a before-after study including 774 brain-injured patients from 20 French ICUs, Asehnoune et al. (2017) reported that full compliance with a ventilatory bundle of low TV (≤7 mL/kg PBW), moderate PEEP (6–8 cm H_2_O), and early extubation was associated with higher ventilator-free days and lower mortality rate at day 90 [132]. However, the optimal ventilatory strategy in patients with ABI, particularly those with TBI, remains controversial [133]. Recently, a systematic review and meta-analysis of eight observational and interventional (before–after) studies including 5639 mechanically ventilated patients with TBI and hemorrhagic stroke reported no significant differences in 28-day or in-hospital mortality, nor in the incidence of ARDS, between patients receiving lung-protective MV and those managed conventionally [66]. Moreover, a recent multicenter RCT (PROLABI trial) comparing low TVventilation (TV = 6 mL/kg PBW) with conventional tidal volume (TV ≥ 8 mL/kg PBW) in patients with ABI found worse outcomes regarding mortality, incidence of ARDS, and ventilator dependency in the lung-protective ventilation group [93]. However, the trial was terminated early due to funding discontinuation, and thus, the generalizability of the results remains uncertain and requires confirmation in larger studies.

Given the uncertainty of the existing data and the lack of high-quality evidence, consensus recommendations for MV in patients with ABI provide only a weak recommendation for the use of lung-protective ventilation, without specifying an exact TV target [12]. Similarly, the Brain Trauma Foundation TBI Guidelines for the Prehospital Management of Traumatic Brain Injury do not provide a recommendation regarding TV and instead suggest maintaining blood oxygen saturation above 90% and ETCO_2_ values between 35 and 45 mm Hg (Figure 2) [101].

## 9. Positive End-Expiratory Pressure

Besides low TV, lung-protective ventilation strategies incorporate the application of PEEP to improve lung compliance and oxygenation by preventing alveolar collapse and recruiting collapsed alveoli, thereby reducing atelectasis [134,135,136,137,138]. Nevertheless, in patients with TBI, particularly those with impaired cerebral autoregulation, the application of PEEP necessitates careful assessment due to the complex interactions with cerebral hemodynamics [82]. Indeed, PEEP may negatively affect both cerebral venous outflow and inflow, leading to alterations in CBF and CBV, and consequently to elevations in ICP [19,53,84,139,140]. These interactions are multifactorial and patient-dependent, influenced by several factors including patient positioning, the level of PEEP, respiratory system mechanics, venous return and cardiac function, baseline ICP and intracranial compliance, and the pressure gradient between ICP and central venous pressure (CVP) [53].

The available research on the effects of PEEP on cerebral dynamics remains controversial. Several studies in brain-injured patients report that increasing PEEP to levels up to 15 cm H_2_O is associated with rises in ICP and reductions in CPP [84,141,142]. This is of particular significance in patients with impaired autoregulation and altered brain compensatory reserves, as ICP can rise above safety limits [83,143]. Additionally, given that the brain is enclosed within the rigid, non-compliant cranial cavity and that the relationship between PEEP elevation and ICP is not linear—each 1 cm H_2_O increase in PEEP resulting in a 0.31 mm Hg increase in ICP—the application of PEEP requires careful consideration [144]. On the other hand, numerous studies suggest that increases in PEEP levels between 5 and 20 cm H_2_O may not impair cerebral parameters requiring clinical intervention if certain conditions are fulfilled, such as normal lung elastance and preserved systemic hemodynamics [84,142,145,146].

Elevation of intrathoracic pressure related to PEEP increases right atrial pressure and consequently CVP, leading to elevations in ICP [147,148]. Li et al. (2020) underscored that the baseline gap between ICP and CVP in mechanically ventilated TBI patients is a strong predictor of the impact of PEEP on ICP, with baseline differences of less than 2.5 mm Hg being associated with a 20% increase in ICP following PEEP elevations up to 15 cm H_2_O [148]. Certainly, according to the Starling resistor model, and given that CVP reflects extracranial venous pressure, PEEP-associated increases in CVP exceeding baseline ICP values decrease venous outflow, thereby elevating ICP [149]. In addition, it has been suggested that PEEP leads to elevations in ICP when it causes alveolar hyperinflation, whereas when it contributes to alveolar recruitment without changes in PaCO_2_, it has no influence on ICP or CPP [150]. Finally, PEEP can impair CBF and CPP by altering systemic hemodynamics, such as cardiac output and arterial blood pressure, particularly in the presence of hypovolemia [143,146], making fluid resuscitation a cornerstone in the management of TBI patients [151]. Interestingly, reductions in MAP and cardiac output can lead to decreases in ICP by lowering CBF and CBV [89].

As previously mentioned, prior research highlights the safety of PEEP application regarding ICP when it enhances alveolar recruitment and lung compliance—even at higher levels—provided there is no hemodynamic compromise. However, the impact of PEEP on parameters such as cerebral autoregulation, which could provide deeper insight into these interactions, remains underinvestigated [84]. Recently, Giardina and colleagues (2023) conducted a prospective observational study involving twenty-five brain-injured patients under multimodal monitoring and MV. They demonstrated that stepwise increases in PEEP from 5 to 15 cm H_2_O did not impair cerebral autoregulation, as assessed by PRx. Although PEEP elevations were associated with significant changes in ICP and CPP, these alterations did not require therapeutic intervention [84].

According to available evidence, Iavarone and co-authors recently suggested a slow and stepwise adaptation of PEEP, not exceeding 5 cm H_2_O, to reach the optimal PEEP level, defined as the level that ensures optimization of systemic and cerebral oxygenation without causing clinically significant alterations in CPP and ICP [82]. The latest ESICM consensus on mechanical ventilation in ABI strongly recommends using a PEEP level similar to that applied in non-brain-injured patients for those with ABI without ARDS who do not have significant ICP elevation or who exhibit PEEP-insensitive ICP elevation. The consensus was unable to recommend a specific PEEP target for patients with ABI without ARDS and intracranial hypertension [12]. A practical approach in the ICU setting involves initiating PEEP at 5 cm H_2_O and gradually titrating it up to 10–15 cm H_2_O under continuous monitoring of cerebral parameters [152,153]. Unfortunately, there is limited evidence on ventilation settings in TBI patients in the prehospital setting [101]. Based on the existing data, we suggest starting with a PEEP level of 5 cm H_2_O [153] while maintaining blood oxygen saturation above 90% and ETCO_2_ values between 35 and 45 mm Hg [101].

## 10. Areas of Future Research

It is well established that MV can induce brain injury in patients without pre-existing brain damage through various pathophysiological mechanisms [154]. Recently, similar to VILI, the concept of ventilator-associated brain injury (VABI) was proposed by Bassi and co-authors (2024), defined as de novo brain dysfunction or damage related to positive-pressure ventilation and not explained by co-existing factors or comorbidities (Figure 1) [80]. Theoretically, VABI may also occur in mechanically ventilated patients with brain injury and could potentially worsen the primary injury or lead to secondary brain damage, resulting in poorer neurological outcomes and prognosis. Therefore, it can be hypothesized that protective MV strategies may influence the development of this phenomenon. This hypothesis warrants clinical attention in patients with TBI, both in the ICU and prehospital settings, and should be validated in diverse clinical scenarios. Moreover, there is insufficient evidence defining optimal ventilatory targets, and future research should aim to improve ventilation algorithms by adjusting tidal volume, PEEP, and respiratory rate guided by ETCO_2_ and SpO_2_, while taking into account brain–lung crosstalk and individualizing strategies for high-risk patients [155].

There is limited evidence regarding the impact of hypoxia and hypercapnia on secondary brain injury. Future research should focus on investigating whether targeted interventions—including non-invasive monitoring, the use of flow-limited bag ventilation devices, or combinations of interventions—can minimize their impact on the development of secondary brain damage [156]. While multimodal neuromonitoring is essential for the management and prognosis of TBI patients [33], its application in the out-of-hospital setting remains very limited. However, non-invasive neuromonitoring technologies, such as near-infrared spectroscopy (NIRS) and point-of-care electroencephalogram (EEG) devices [157,158], could contribute to prehospital management and guide therapeutic interventions, including ventilatory strategies. Further research is needed to evaluate their diagnostic value and potential to inform treatment decisions.

Finally, there is a lack of randomized controlled trials evaluating optimal airway management, with reported outcomes varying between positive and negative. Given the many hypothesized contributing factors—such as hypoxic episodes during airway management, malpositioned or ineffective advanced airways, and hyperventilation during MV [159]—future research should address these issues and determine the optimal advanced airway intervention (e.g., endotracheal intubation, supraglottic airways, or bag-valve-mask ventilation), tailored to the individual patient.

## 11. Limitations of the Existing Evidence

Several important limitations characterize the existing evidence. Most of the available studies are retrospective observational analyses and therefore provide primarily Class III evidence [160]. Moreover, their designs are highly heterogeneous and subject to various sources of bias, frequently involving small sample sizes that do not allow for multivariate analyses, missing data, and limited internal validity [101,161]. In many cases, studies rely on historical controls or inadequately described comparison groups. In addition, long-term outcomes are insufficiently reported [161]. Well-designed, high-quality randomized clinical trials are required to determine the best strategies for optimizing patients’ outcomes.

## 12. Conclusions

MV in patients with TBI poses a unique challenge in optimizing oxygenation, preventing secondary brain injury, and managing extracranial organ dysfunction while considering various factors such as cerebral autoregulation and autoregulatory reserve, cerebral dynamics including ICP, CPP, and CBF, brain compliance, systemic hemodynamics, and respiratory parameters. However, strong evidence to define the appropriate ventilatory strategy in these patients is lacking, particularly regarding lung-protective MV, which may lead to hypercapnia and an associated rise in ICP. Moreover, the application of PEEP, a main element of lung-protective ventilation, is associated with reduced cerebral venous outflow, hemodynamic compromise, decreased CPP, and elevation of ICP. As pulmonary complications and secondary brain injury can begin shortly after the traumatic event, prehospital mechanical ventilation (MV) plays a fundamental role in patient outcomes. Despite the scarcity of existing data, in the out-of-hospital setting, patients with TBI should be managed according to existing consensus recommendations, with basic ventilatory settings including a TV of 6–8 mL/kg of PBW, PEEP of 5 cm H_2_O, and a respiratory rate of 16–22 breaths per minute, targeting an arterial oxygen saturation above 90% and an ETCO_2_ value between 35 and 45 mm Hg.

## Data Availability

No new data were created or analyzed in this study.
